# Pathogenic gene variants in *CCDC39*, *CCDC40*, *RSPH1*, *RSPH9*, *HYDIN,* and *SPEF2* cause defects of sperm flagella composition and male infertility

**DOI:** 10.3389/fgene.2023.1117821

**Published:** 2023-02-17

**Authors:** I. Aprea, A. Wilken, C. Krallmann, T. Nöthe-Menchen, H. Olbrich, N. T. Loges, G. W. Dougherty, D. Bracht, C. Brenker, S. Kliesch, T. Strünker, F. Tüttelmann, J. Raidt, H. Omran

**Affiliations:** ^1^ Department of General Pediatrics, University Hospital Münster, Münster, Germany; ^2^ Department of Clinical and Surgical Andrology, Centre of Reproductive Medicine and Andrology, University Hospital Münster, Münster, Germany; ^3^ Centre of Reproductive Medicine and Andrology, University Hospital Münster, University of Münster, Münster, Germany; ^4^ Institute of Reproductive Genetics, University of Münster, Münster, Germany

**Keywords:** axonemal ruler, RS head, CP-complex, PCD, asthenozoospermia, MMAF

## Abstract

Primary Ciliary Dyskinesia (PCD) is a rare genetic disorder affecting the function of motile cilia in several organ systems. In PCD, male infertility is caused by defective sperm flagella composition or deficient motile cilia function in the efferent ducts of the male reproductive system. Different PCD-associated genes encoding axonemal components involved in the regulation of ciliary and flagellar beating are also reported to cause infertility due to multiple morphological abnormalities of the sperm flagella (MMAF). Here, we performed genetic testing by next generation sequencing techniques, PCD diagnostics including immunofluorescence-, transmission electron-, and high-speed video microscopy on sperm flagella and andrological work up including semen analyses. We identified ten infertile male individuals with pathogenic variants in *CCDC39* (one) and *CCDC40* (two) encoding ruler proteins, *RSPH1* (two) and *RSPH9* (one) encoding radial spoke head proteins, and *HYDIN* (two) and *SPEF2* (two) encoding CP-associated proteins, respectively. We demonstrate for the first time that pathogenic variants in *RSPH1* and *RSPH9* cause male infertility due to sperm cell dysmotility and abnormal flagellar RSPH1 and RSPH9 composition. We also provide novel evidence for MMAF in *HYDIN*- and *RSPH1*-mutant individuals. We show absence or severe reduction of CCDC39 and SPEF2 in sperm flagella of *CCDC39*- and *CCDC40*-mutant individuals and *HYDIN*- and *SPEF2*-mutant individuals, respectively. Thereby, we reveal interactions between CCDC39 and CCDC40 as well as HYDIN and SPEF2 in sperm flagella. Our findings demonstrate that immunofluorescence microscopy in sperm cells is a valuable tool to identify flagellar defects related to the axonemal ruler, radial spoke head and the central pair apparatus, thus aiding the diagnosis of male infertility. This is of particular importance to classify the pathogenicity of genetic defects, especially in cases of missense variants of unknown significance, or to interpret *HYDIN* variants that are confounded by the presence of the almost identical pseudogene *HYDIN2*.

## Introduction

Infertility affects about 15% of couples, with male infertility accounting for half of the cases. Male infertility is a multifactorial condition that includes quantitative and qualitative sperm defects with various causes such as infectious, hormonal or genetic. The cause of many cases of male infertility remains unexplained, but genetic defects have been increasingly discovered in recent years (Bracke et al., 2018; Tüttelmann et al., 2018; Sironen et al., 2019; Aprea et al., 2021b; Touré et al., 2021). A heterogeneous group of genetic disorders caused by dysfunction of motile cilia in various organ systems, termed motile ciliopathies, plays an important role in the etiology of male infertility (Wallmeier et al., 2020). The most common motile ciliopathy is the multisystem disorder primary ciliary dyskinesia (PCD; MIM244400), which is characterized by recurrent respiratory symptoms. Dyskinetic motile cilia of the upper and lower airways can impair mucociliary clearance and result in a chronic destructive airways disease. Motility of monocilia at the embryonic node is essential for the correct establishment of the left–right body asymmetry. Failure to establish the typical arrangement of visceral organs (*situs solitus*) manifests in laterality defects, such as the mirrored arrangement of organs (*situs inversus totalis*), or heterotaxy (*situs ambiguous*), which includes all other types of laterality defects (e.g., *situs inversus* of just one body cavity) and is often associated with congenital heart defects. In rare cases ependymal cilia of the brain ventricles contribute to the formation of hydrocephalus, that represents the condition of dilated brain ventricles due to cerebrospinal fluid accumulation (Wallmeier et al., 2019). Reduced female and male fertility is also observed in motile ciliopathies, due to the dysfunction of fallopian tube cilia or the dysmotility of sperm flagella (Wallmeier et al., 2020). Recently, our group identified a novel ciliary disease mechanism that affects male fertility by impairing motile cilia in the male reproductive efferent ducts. We demonstrated that dysmotility of efferent duct cilia can cause reduced sperm count (oligozoospermia) due to obstructive sperm stasis in these extra-testicular tubules (Aprea et al., 2021a). Most motile ciliopathies are caused by autosomal recessive inherited mutations, but less frequently, X-chromosomal recessive or autosomal dominant inheritance patterns are observed (Olbrich et al., 2002; Omran et al., 2008; Paff et al., 2017; Loges et al., 2018; Wallmeier et al., 2019; Wallmeier et al., 2020). So far, more than 50 genes are known to be associated with motile ciliopathies and the clinical features of motile ciliopathies vary depending on whether the distinct gene defect affects only 1 cell type or several cell types in different combinations (Wallmeier et al., 2020).

**FIGURE 1:**
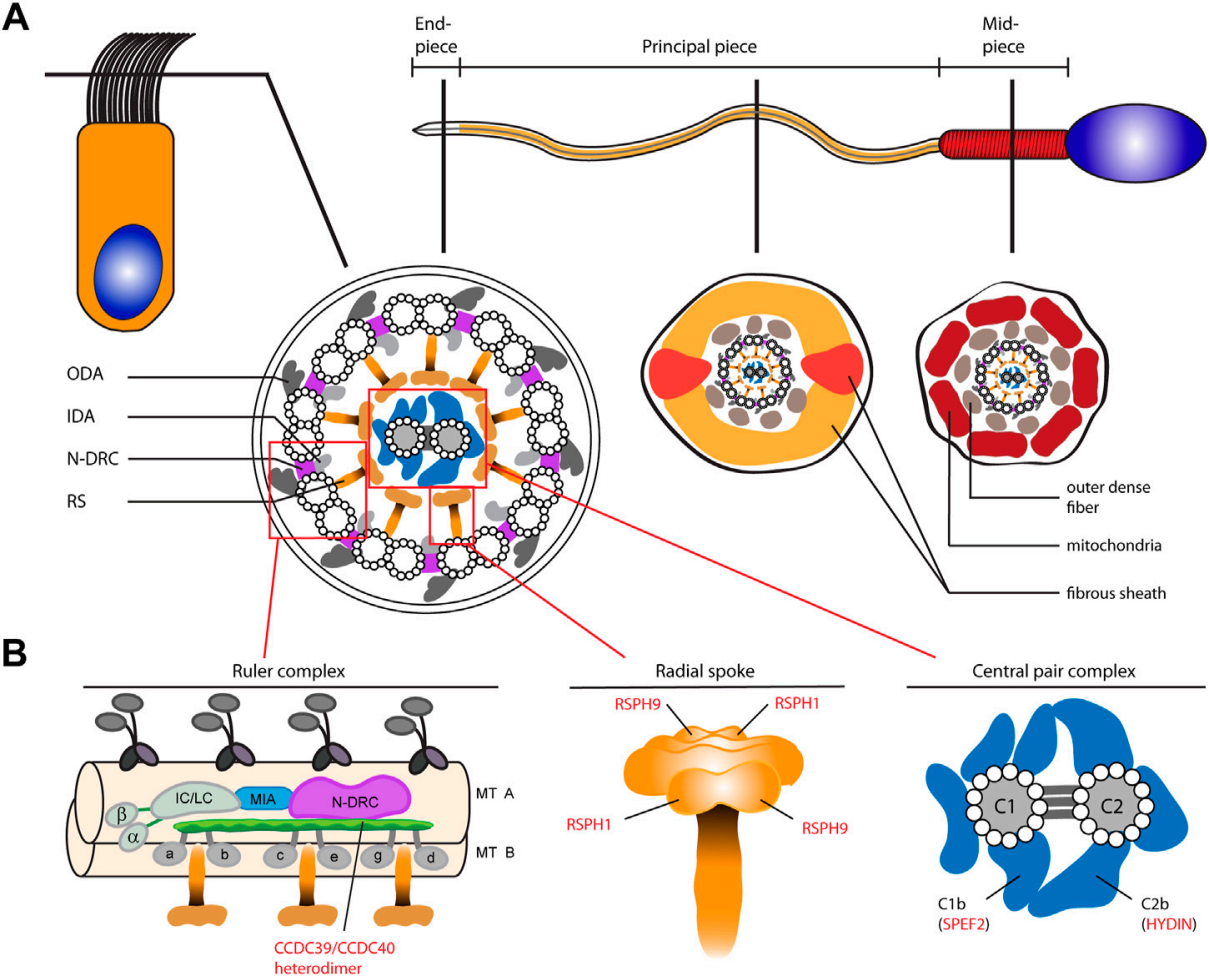
Schematic of the composition of motile 9 + 2 cilia and sperm flagella as well as representation of selected functional axonemal protein complexes involved in ciliary and flagellar beat regulation. **(A)** The upper panel shows a schematic representation of a ciliated respiratory epithelial cell (left) and a sperm cell (right). The sperm flagellum is divided into three regions; the mid piece, principal- and end piece. The middle panel shows cross sections through a respiratory cilia and the sperm flagellum. Both share a 9 + 2 axoneme composed of nine outer microtubule doublets (A- and B-tubule) surrounding a centered single pair of microtubules. Each outer A-tubule carries dynein based motor protein complexes, the outer- and inner dynein arms (respectively ODA and IDA). A nexin-link dynein regulatory complex (N-DRC) interconnects the outer doublets, while radial spoke (RS) complexes connect them to the central pair (CP) complex. In the sperm flagellar principal- and mid-piece the axoneme is additionally surrounded by accessory structures, such as the outer dense fibers (ODFs), the fibrous sheath (FS) and the mitochondrial (MT) sheath. **(B)** The lower panel shows schematics of the axonemal ruler, the RS and the CP complex. The ruler complex is representatively depicted in a longitudinal section of the axoneme. CCDC39 and CCDC40 form a heterodimer that is supposed to be located at the interface of the N-DRC and IDAs (composed of double headed IDA I1 with heavy chains α and β and the intermediate- and light chain (IC/LC) complex, and single headed IDAs a-e, and g) (Olbrich et al., 2015; Yamamoto et al., 2021). The RS complex is composed of a stalk and a head structure. RSPH1 and RSPH9, here highlighted, are known to be components of the RS head in human respiratory cilia (Frommer et al., 2015). The CP-complex is composed of two central microtubules connected by three intermicrotubular bridges. Several protein complexes are associated with the CP, e.g., the C1b and C2b projections, containing the proteins SPEF2 and HYDIN, respectively. In human respiratory cilia localization of SPEF2 depends on functional HYDIN (Cindrić et al., 2019).

Motile cilia and sperm flagella share an evolutionarily conserved microtubule-based ultrastructure with nine outer doublets surrounding a pair of two single central tubules (9 + 2 axoneme; central pair (CP)). The core component is a 9 + 2 axoneme with several associated functional protein complexes that generate and regulate ciliary and flagellar beating, such as the outer dynein arms (ODAs), the axonemal ruler, the radial spokes (RSP), and the CP (Figure 1A) (Wallmeier et al., 2020). As sperm flagella resemble the structure of 9 + 2 multiple motile cilia in the airways, molecular defects in respiratory cilia can also result in impaired sperm motility. Thus, some defects of axonemal structures associated with ciliary and flagellar beat regulation are known to cause both PCD (respiratory cilia dysfunction) and male infertility due to multiple morphological abnormalities of the sperm flagella (MMAF, sperm flagellar dysfunction). These include pathogenic variants in genes encoding the axonemal ruler proteins CCDC39 and CCDC40 (Figure 1B), or the CP apparatus (Figure 1B) related gene *SPEF2* (Becker-Heck et al., 2011; Merveille et al., 2011; Antony et al., 2013; Cindrić et al., 2019; Sha et al., 2019; Chen et al., 2021; Xu et al., 2022). However, because respiratory cilia and sperm flagella do not share all proteins, some male individuals with PCD can father children without assisted reproduction. For example, individuals with mutations in *CCDC114* encoding an ODA docking complex protein only suffer from PCD without male infertility, because CCDC114 is not present in sperm flagella (Knowles et al., 2013). In addition, several gene defects cause isolated defects of the sperm flagellum. For example, mutations in *DNAH8* and *DNAH17*, encoding sperm specific ODA heavy chains, result in immotile sperm tails and multiple morphological abnormalities of sperm flagella, without any respiratory cilia dysfunction (Whitfield et al., 2019; Liu et al., 2020). For many motile ciliopathies it is unknown if the defect is affecting respiratory cilia alone or in conjunction with sperm flagellar defects. For instance, defects in axonemal radial spoke head proteins RSPH1 and RSPH9 (Figure 1B), which also regulate ciliary beat, affect composition and function of respiratory cilia but it is unknown whether sperm flagella are also affected (Frommer et al., 2015).

However, the disease mechanisms underlying MMAF and male infertility are still not fully understood and only few cases have been reported. Detailed molecular and cell biological investigations of mutant sperm flagella are lacking.

In this study, we identified individuals with pathogenic variants in *CCDC39* and *CCDC40* (encoding for components of the axonemal ruler), *RSPH1* and *RSPH9* (encoding radial spoke head proteins), as well as *HYDIN* and *SPEF2* (encoding components of the CP associated apparatus 1B and 2B, respectively) (Figure 1B). We previously demonstrated in respiratory cilia that CCDC39 and SPEF2 localization are dependent on functional CCDC40 and HYDIN, respectively (Becker-Heck et al., 2011; Merveille et al., 2011; Antony et al., 2013; Cindrić et al., 2019), and that RSPH1 and RSPH9 are both components of the radial spoke head structure (Figure 1B) (Frommer et al., 2015). We hypothesized that these mutations would cause similar axonemal defects in sperm flagella. To determine if these affected individuals also have defective sperm flagella composition and function (Figure 1B), and thus clarify if the analyzed proteins are only involved in function of motile cilia or also play a role in sperm flagella beat regulation, we performed spermiograms, high speed video microscopy analyses (HVMA), immunofluorescence (IF) microscopy and transmission electron microscopy (TEM) of sperm cells.

Here, we demonstrate that defects in the respective genes cause abnormal composition of the axonemal ruler, radial spoke head and CP-apparatus, also in sperm flagella. The effects of all reported genetic variants on encoded proteins can be detected by immunofluorescence microscopy (IF) not only in respiratory epithelial cells but also in sperm flagella. These findings can therefore aid the diagnosis in male infertility.

## Materials and methods

### Ethic statement

This research project was approved by the Ethics Committee of the Medical Association of Westphalia-Lippe and the Westphalian Wilhelms University of Münster (Ärztekammer Westfalen Lippe and Westfälische Wilhelms Universität; reference number: AZ 2017-139-f-S; 2011–270-f-S, 2010- 578-f-S; 4INie). Prior to participation, each participating individual and family members gave written informed consent.

### Study cohort

The fertility status as well as composition of sperm flagella and respiratory motile cilia from ten adult males either diagnosed with PCD (OP-individuals) or MMAF associated infertility (SP-individuals), and confirmed by disease causing mutations in PCD- and MMAF associated genes, were examined. Based on gender, age (at least 18 years of age) and genetic defect in a known PCD- and/or MMAF causing gene, eight PCD individuals were selected from the cohort of the University Childrens Hospital, Münster (Germany), and two MMAF affected individuals from the cohort of the Centre of Reproductive Medicine and Andrology (CeRA), University Hospital, Münster (Germany). Examinations included andrological examination with semen analyses at the CeRA and PCD diagnostic work up at the University Childrens Hospital. Systematic investigation of distinct axonemal components was performed in sperm flagella and respiratory epithelial cells.

### Human samples

For this study, we collected ciliated respiratory epithelial cells from nasal brush biopsies and sperm cells from donated ejaculate from ten PCD-affected men and respective healthy control individuals. Samples were used for high-resolution IF microscopy, HVMA and when applicable for TEM. For the isolation of DNA used for genetic testing, we collected peripheral blood from participating individuals.

### Semen analyses

Semen analyses were performed in the andrology laboratory of the Centre for Reproductive Medicine and Andrology (CeRA), University Hospital, Münster (Germany), according to latest WHO guidelines (World Health Organization, 2010). Participating individuals donated semen after 2–7 days of sexual abstinence. After liquefaction for 30 min at 37°C their semen was analyzed for sperm concentration and motility, as well as vitality and morphology. Additionally, the ejaculate of each individual was used for IF microscopy, HVMA and TEM, when applicable.

### Genetic testing

Genomic DNA was isolated from collected blood samples using standard methods. To assess the genetic cause in PCD-affected individuals with undiagnosed mutational status, we performed a customized PCD gene panel (comprising known PCD-associated genes, as well as candidate genes), whole-exome sequencing (WES) and Sanger sequencing, as previously described (Höben et al., 2018; Loges et al., 2018; Aprea et al., 2021b). WES of genomic DNA was performed at the Cologne Center for Genomics. Briefly, target DNA capture was performed using the NimbleGen SeqCap EZ Human Exome Library v2.0 and enriched preparations sequenced with the HiSeq2000 platform (Illumina). Sequencing reads that passed quality filtering were mapped to the human reference genome hg38. The infertile males selected from the CeRA-cohort underwent WES in the Male Reproductive Genomics (MERGE) study at the Institute of Reproductive Genetics, University of Münster, Germany as described previously (Wyrwoll et al., 2021). Sample preparation and enrichment for exome sequencing were performed using either Agilent’s SureSelectQXT Target Enrichment kit or Twist Bioscience’s Twist Human Core Exome kit. Quantity and quality of the libraries were assessed using the ThermoFisher Qubit and Agilent’s TapeStation 2200, respectively. Enriched preparations were sequenced on the Illumina HiScan®SQ, NextSeq^®^500/550 or HiSeqX^®^ systems using the TruSeq SBS Kit v3-HS (200 cycles), the NextSeq 500 V2 High-Output Kit (300 cycles) or the HiSeq Rapid SBS Kit V2 (300 cycles), respectively. Sequence reads were aligned against the reference genome GRCh37. p13 using BWA Mem v0.7.17.

We filtered for non-synonymous mutations, splice-site substitutions, and indels following an autosomal- and X-linked recessive inheritance pattern and excluded variants present in the dbSNP database, the 1000 Genomes Project polymorphism, and the Genome Aggregation Database (gnomAD) (Karczewski et al., 2020) with a minor-allele frequency of >0.01. Detected genetic variants were evaluated according to international guidelines (Richards et al., 2015). The predicted consequence and classification of pathogenicity of splice-site variants was analyzed using varSEAK (www.varSEAK.bio, developed by JSI medical systems GmbH, Ettenheim, Germany). The predicted pathogenicity of missense variants was evaluated using several programs, including SIFT, PROVEAN, Mutation Taster and PolyPhen (Adzhubei et al., 2010; Schwarz et al., 2014; Choi and Chan, 2015; Vaser et al., 2016; Kopanos et al., 2019). Primer sequences used for variant verification by Sanger Sequencing are available in Supplementary Table S1.

### Immunofluorescence microscopy (IF)

Respiratory and sperm cell samples were treated and incubated with primary and secondary antibodies as reported in previous studies (Omran and Loges, 2009; Cindrić et al., 2019; Aprea et al., 2021a). Antibodies and their applied dilutions are listed in Supplementary Table S2. Cell nuclei were stained using Hoechst33342 (Thermo Fischer Scientific, Waltham, United States). Samples were mounted in DAKO fluorescence mounting medium (Dako North America Inc., Carpinteria, United States). IF images were taken with a Zeiss AxioObserver Z1 Apotome (Carl Zeiss Meditec AG, Oberkochen, Germany) or Laser Scanning Microscope LSM 880 (Carl Zeiss Microscopy GmbH, Jena, Germany). Images were processed and exported using the ZEISS ZEN Imaging Software 2012 (Carl Zeiss Microscopy GmbH, Jena, Germany) and figure panels created with the Adobe Creative Suite CS5 (Adobe Systems, San José, United States).

### High speed video microscopy analysis (HVMA)

High-speed video microscopy analysis of respiratory cilia and assessment of ciliary beat frequency and pattern was performed as previously described (Raidt et al., 2014), using an Eclipse Ti Inverted Microscope (Nikon, Tokyo, Japan) connected to an acA1300-200um - Basler ace camera (Basler AG, Ahrensburg, Germany). Sperm flagella beating was assessed with 10 µL liquefied ejaculate at ×20 and ×40 magnification at 37°C.

### Transmission electron microscopy (TEM)

Nasal brush biopsies and 250 µL of liquefied ejaculate were each fixed in 2.5% glutaraldehyde over night at 4°C. Cell samples were pelleted, washed with tap water, and then incubated at room temperature for 1.5–2 h in 1% osmium tetroxide. After dehydration in an ethanol series, samples were transferred to 1, 2-epoxypropan first and then incubated in a 1, 2-epoxypropan-epon mixture (1:2) at 4°C overnight. Finally, samples were embedded in epon and dried at 65°C. Ultrathin sections (80 nm) of samples were analyzed with the transmission electron microscope Philips CM10 and TEM images acquired with a Quemesa camera and the iTEM SIS image acquisition software (both from Olympus Soft Imaging Solutions). Image processing was performed using Adobe Creative Suite CS5.

### Nasal nitric oxide (nNO)-production rates

As recommended by the American Thoracic- and European Respiratory Society (ATS/ERS recommendations for standardized procedures for the online and offline measurement of exhaled lower respiratory nitric oxide and nasal nitric oxide, 2005, American Thoracic Society and European Respiratory Society, 2005), nasal nitric oxide (nNO)-production rates are determined as part of the PCD routine diagnostic setup. Assessment of the nNO-production rates were performed as previously described (Werner et al., 2015; Raidt et al., 2022). Specifically, a chemiluminescence analyzer was used according to current PCD diagnostic guidelines (Lucas et al., 2017). All patients were able to perform a velum closure technique.

## Results

A next-generation sequencing approach using a targeted PCD gene panel and WES in eight genetically undiagnosed males with suspected PCD and infertility revealed pathogenic variants in *CCDC40*, *RSPH1*, *RSPH9*, *HYDIN,* and *SPEF2*. In these individuals and two other males that were previously identified with pathogenic variants in *CCDC39* and *HYDIN* (Merveille et al., 2011; Olbrich et al., 2012), further molecular and cell biological analyses were performed to characterize in more detail the composition and function of the axonemal ruler, RS and CP complex in sperm flagella.

### Pathogenic variants in *CCDC39* and *CCDC40* cause absence of the axonemal ruler component CCDC39 from sperm flagellar axonemes and lead to MMAF-related infertility

So far, the molecular defects in sperm flagella of *CCDC39*- and *CCDC40*-mutant males have not been characterized in detail. Here we had the opportunity to address this important research question.

Using a targeted PCD gene panel (OP-1516 II1) and WES (OP-11 II2) in two PCD-affected males we identified pathogenic variants in *CCDC40* (CCDS42395; GenBank: NM_017950.4). In individual OP-11 II2, we diagnosed a homozygous transition leading to a premature termination of translation: c.1675G>T (p.Glu559*). Sanger sequencing confirmed the same homozygous variant in the affected sister OP-11 II1 (Supplementary Figure S1A). In individual OP-1516 II1, we identified the previously reported point deletion c.248del (rs397515393) (p.Ala83Valfs*84) (Becker-Heck et al., 2011; Antony et al., 2013), and the insertion c.736_755dup (rs753711384) (p.Ser252Argfs*43), both leading to a frameshift and premature translational stop. Sanger sequencing identified the variant c.248delC in the mother OP-1516 I2, and variant c.736_755dup in the father OP-1516 I1 (Supplementary Figure S1A) confirming the compound heterozygous status in OP-1516 II1. According to the guidelines of the American College of Medical Genetics (ACMG) (Richards et al., 2015), *CCDC40* variants c.1675G>T and c.736_755dup are classified as likely pathogenic (class 4), and variant c.248delC as pathogenic (class 5).

Consistent with previous studies in individuals with defects of the axonemal ruler (Merveille et al., 2011; Antony et al., 2013) *CCDC40*-mutant individual OP-11 II2 presented with *situs inversus totalis* and bronchiectasis. Moreover, the patient reported fertility issues. Individual OP-1516 II1 reported neonatal respiratory distress syndrome (RDS) and was diagnosed with nNO-production rates below the current cutoff value of 77 nL/min, recurrent otitis media, sinusitis, pneumonia and lung disease (Table 1). Semen analyses in OP-1516 II1 revealed asthenozoospermia due to immotile sperm (Supplementary Video S2), with additionally an abnormal sperm morphology and a reduced sperm count (Table 1). A retrospective analysis of the fertility status of *CCDC39*-mutant individual OP-122, whose genetic and clinical findings were reported previously (Merveille et al., 2011) and are summarized in Table 1, determined that OP-122 presents also with immotile sperm cells (Supplementary Video S3 and Table 1).

**TABLE 1:** Clinical characteristics of study participants. aa: amino acids; Chron: chronic; del: deletion; dup: duplication; FEV: forced expiratory volume; hom: homozygous; ml: milliliter; N/A: not annotated; n.a: not available; nl: nanoliter; nNO; nasal nitric oxide-production rate; RDS: respiratory distress syndrome, ref: reference value.

	Ruler machinery mutant individuals			RS mutant individuals					CP mutant individuals				
	OP-122	OP-11 II2	OP-1516 II1	OP-3257 II1		OP-3957 II1	OP-2491 II1		OP-305 II2	OP-3022 II2	SP-24/M2321	SP-32/M2334	
Mutated gene	*CCDC39*	*CCDC40*	*CCDC40*	*RSPH1*		*RSPH1*	*RSPH9*		*HYDIN*	*HYDIN*	*SPEF2*	*SPEF2*	
cDNA mutation	c.1072del	c.1675G>T (hom.)	c.248del	c.680dup (hom.)		c.501+2dup (hom.)	c.244T>C (hom.)		c.3984 + 1G>T (hom.)	c.6140C>G (hom.)	c.910C>T	c.2763-2764del	
	c.1007-1010del		c.736_755dup								c.2629del	c.3620G>A	
Protein alteration	(p.Thr358Glnfs*3)	(p.Glu559*)	(p.Ala83Valfs*84)	(p.Pro228Alafs*15)		(p.?)	(p.Trp82Arg)		frameshift-stop after insertion of 62 novel aa	(p.Ser 2047*)	(p.Arg304*)	(p.Glu921Aspfs*31)	
	(p.Lys336Argfs*19)		(p.Ser252Argfs*43)								(p.Ile877Phefs*6)	(p.Arg1207Lys)	
Mutation type	frameshift	non-sense	frameshift	frameshift		obligatory splicesite	missense		obligatory splicesite	non-sense	non-sense	frameshift	
	frameshift		frameshift								frameshift	missense	
Allele Frequency (GnomAD)	3.25e-5	N/A	4.27e-4	2.48e-5		3.98e-6	N/A		N/A	N/A	7.97e-6	N/A	
	N/A		8.04e-6								N/A	5.87e-5	
Age at semen analysis	n.a	n.a	24	27	28	24	40	40	n.a	25	31	37	
Semen volume (ml), ref: ≥1.5	n.a	n.a	3.0	3.4	2.3	2.9	4.4	5.2	n.a	2.0	6.4	4.1	
Total sperm number (10^6^), ref: ≥39	n.a	n.a	17.7	295.1	227.2	16.5	456.5	313.6	n.a	97.6	137	70.5	
Sperm concentration (10^6^/ml), ref: ≥15	n.a	n.a	5.9	86.8	98.8	5.70	103.8	60.3	n.a	48.8	21.4	17.2	
Progressive motility (PR, %), ref: ≥32	n.a	n.a	0	21	13	22	2	0	n.a	0	0	1	
Sperm morphology (normal forms, %), ref: ≥4	n.a	n.a	1	4	n.a	1	38	36	n.a	0	2	3	
Vitality (live spermatozoa, %), ref: ≥58	n.a	n.a	75	52	56	72	2	0	n.a	72	72	65	
pH, (ref: ≥7.2)	n.a	n.a	8.5	8.5	8.3	7.9	7.4	7.4	n.a	8.3	8.3	8.1	
RDS	yes	n.a	yes	no		yes	n.a		yes	yes	no	no	
Chron. middle ear symptoms	n.a	n.a	yes	no		yes	yes		yes	yes	yes	no	
Chron. sinusitis/rhinitis	yes	n.a	yes	yes		yes	yes		yes	yes	yes	yes	
Chron. bronchitis/pneumonia, wet cough	yes	n.a	yes	yes		yes	yes		yes	yes	no	no	
Bronchiectasis	yes	n.a	yes	yes		yes	yes		yes	yes	n.a	n.a	
Situs	abdominalis/ambiguus	inversus totalis	solitus	solitus		solitus	solitus		solitus	solitus	solitus	solitus	
FEV1	18.5%	n.a	76.4%	96.1%		94.9%	47.6%		n.a	61%	99.1%	93%	
nNO (nL/min)	n.a	n.a	30	149		21	18		n.a	19	226	62	

**FIGURE 2:**
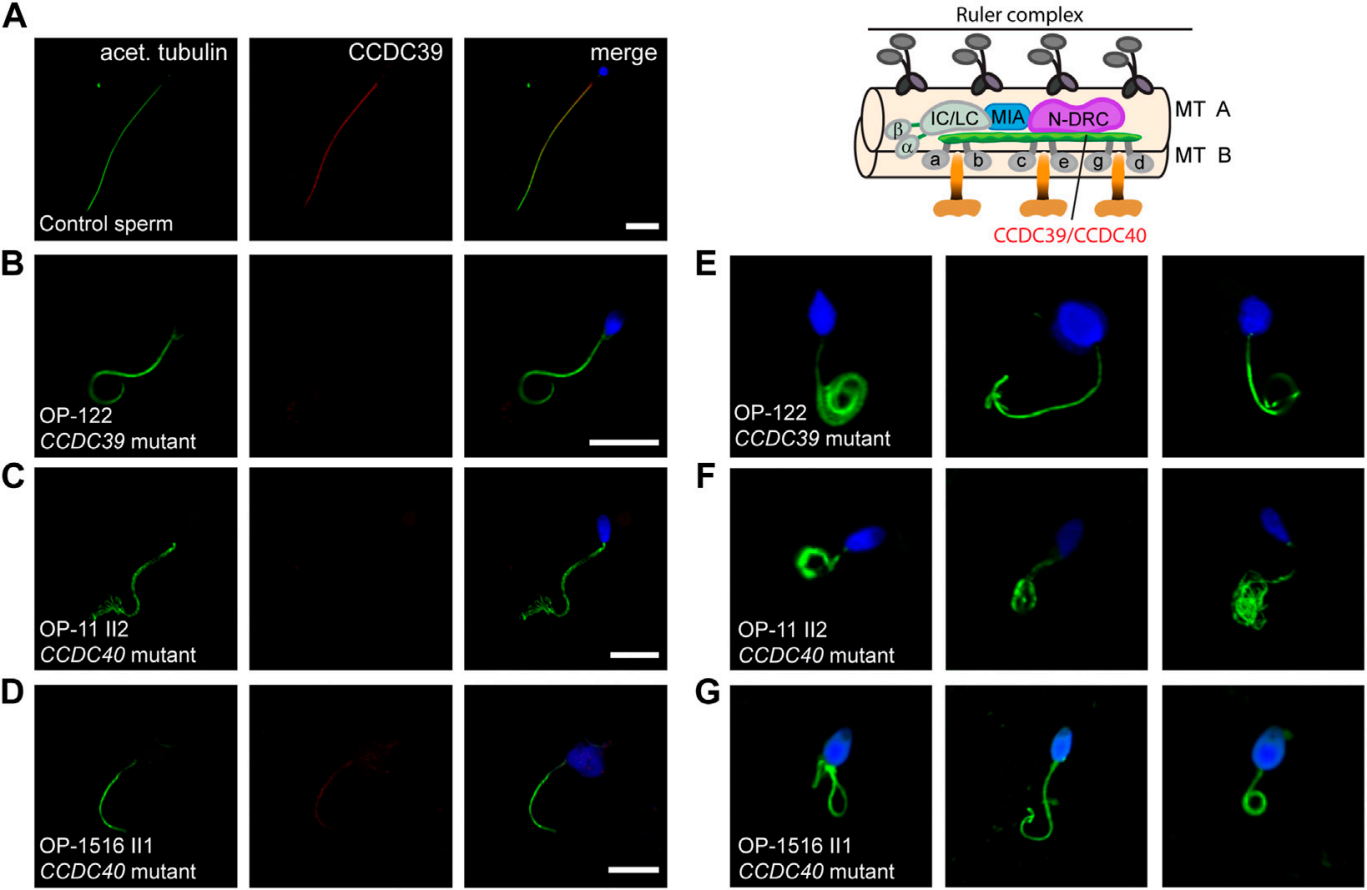
*CCDC40*-mutant individuals display MMAF and CCDC39 is absent from CCDC39- and CCDC40-mutant sperm flagellar axonemes. Sperm cells from healthy control donors and infertile male individuals with pathogenic variants in CCDC39 and CCDC40 were co-stained with antibodies directed against acetylated α-tubulin (acet. tubulin; depicted in green) and CCDC39 (depicted in red). **(A)** In control sperm, immunoreactivity against CCDC39 (red) is observed along the entire flagella length. The CCDC39 signal co-localizes with the flagellar marker acet. tubulin (depicted in yellow in the merged image). **(B–D)** In sperm flagella of *CCDC39*-mutant individual OP-122 and *CCDC40*-mutant individuals OP-11 II2 and OP-1516 II1, no signal against CCDC39 is observed along the flagellar axoneme. **(E–G)** Sperm cells of *CCDC39*-mutant individual OP-122 and *CCDC40*-mutant individuals OP-11 II2 and OP-1516 II1 display morphological abnormalities, comprising short, bend and irregular flagella, as well as head defects. The schematic representatively shows the localization of the CCDC39/CCDC40 heterodimer. Nuclei were stained with Hoechst33342. Scale bars represent 10 µm.

We have previously shown that the ruler protein CCDC39 is absent from *CCDC39*- and *CCDC40*-mutant respiratory cilia (Becker-Heck et al., 2011; Merveille et al., 2011; Antony et al., 2013). Consistently, we found absence of CCDC39 from the whole ciliary axonemes (Supplementary Figure S2) in individuals OP-122 and OP-1516 II1, which confirms the pathogenicity of the identified variants at the protein level. So far, CCDC39 localization has not been studied in *CCDC40*-mutant sperm flagella. First, we analyzed *CCDC39* localization in *CCDC39*-mutant sperm flagella. In individual OP-122 with pathogenic variants in *CCDC39*, co-staining against the flagellar marker acetylated α-tubulin and CCDC39 showed that sperm flagella of all affected males indeed lack CCDC39 along the entire flagella length (Figures 2A, B) confirming that our IF technique can also be applied in sperm flagella. Next, we demonstrate that the axonemal ruler protein CCDC39 is also absent from flagellar axonemes of *CCDC40*-mutant individuals (OP-11 II2 and OP-1516 II1, Figures 2C, D). Thus, our findings demonstrate for the first time that CCDC39 and CCDC40 also interact in sperm flagella. In addition, we demonstrate that the pathogenicity of *CCDC39* and *CCDC40* variants can be also established by IF analyses in sperm cells.

**FIGURE 3:**
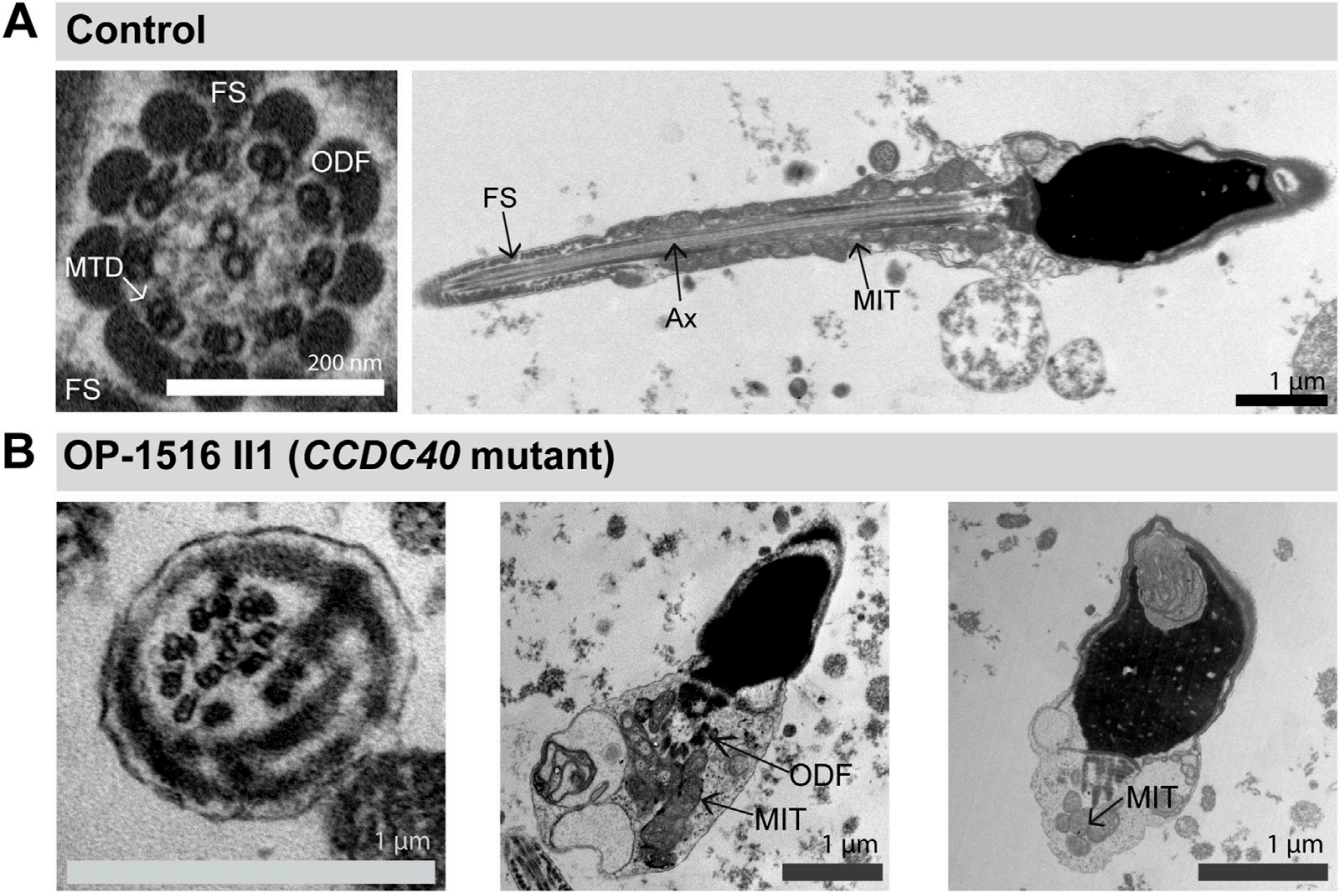
TEM analyses of *CCDC40*-mutant sperm cells show morphological abnormalities and tubular disorganisation. **(A)** TEM micrographs of sperm samples from a healthy control donor, showing a flagellar cross section with a regular 9 + 2 axoneme of microtubule doublets (MTD), (left image), and a longitduinal section showing the axoneme (Ax) at the center, with regularly composed mitochondria (MIT) and fibrous sheath (FS), respectively in the flagellar mid piece and principal piece (right image). **(B)** *CCDC40-*mutant individual OP-1516 II1 shows morphological abnormalities of the sperm cells. Some sperm cells depict severe head malformations and lack proper flagella, displaying a cytoplasmic bag with unassembled peri-axonemal components, such as outer dense fibers (ODF) and mitochondria (right and middle image). In sperm flagella cross sections, axonemes show severe tubular disorganization (left image).

Consistent with a reduced percentage of spermatozoa with abnormal morphology observed in the semen analysis of OP-1516 II1 and with previous findings (Chen et al., 2021; Liu et al., 2021; Xu et al., 2022), we found by IF microscopy different morphological abnormalities in sperm cells of *CCDC39*- and *CCDC40*-mutant males, including short, bend and irregular flagella, as well as sperm head defects typical for MMAF (Figures 2E–G). To further characterize the sperm flagellar phenotype, we performed ultrastructural analyses of semen from *CCDC40*-mutant male OP-1516 II1. We identified classical findings of MMAF, including sperm with cytoplasmic bags containing unassembled axonemal and peri-axonemal components, as well as severe tubular disorganization of the flagellar axoneme resembling reported findings of axonemal disorganization in respiratory cilia (Figure 3).

### Disease-causing variants in *RSPH1* and *RSPH9* result in altered radial spoke head composition in sperm flagella and cause male infertility due to dysmotile sperm cells

So far, the molecular defects in sperm flagella of *RSPH1*- and *RSPH9*-mutant males have not been characterized previously. Here we had the opportunity to address this important research question.

In three PCD-affected male individuals we diagnosed pathogenic variants in two genes encoding different components of the RS head. In OP-3257 II1 and OP-3957 II1, we detected homozygous variants in *RSPH1* (CCDS13688; GenBank: NM_080860.4). In OP-3257 II1, we found the insertion c.680dup (rs556286752) (p.Pro228Alafs*15), leading to a frameshift and premature translational stop (ACMG classification: class 5). For OP-3957 II1, we detected a donor splice-site variant c.501+2dup (rs1568964336) in intron 5-6 strongly predicted to activate a cryptic splice site located 28 nucleotides upstream in exon 5 and therefore pathogenic (varSEAK classification: class 5). Sanger sequencing confirms segregation in both families (Supplementary Figure S1B). Individual OP-2491 II1 was diagnosed with the homozygous missense variant c.244T>C (p.Trp82Arg) in *RSPH9* (CCDS4905; GenBank: NM_152732.5) (Supplementary Figure S1B). This variant is predicted to be damaging by several prediction programs, including SIFT, PROVEAN, Mutation Taster and PolyPhen (Adzhubei et al., 2010; Schwarz et al., 2014; Choi and Chan, 2015; Vaser et al., 2016; Kopanos et al., 2019).

Individuals OP-3257 II1 and OP-3957 II1, with pathogenic variants in *RSPH1,* both displayed chronic respiratory symptoms including chronic otitis media, sinusitis, bronchitis, and chronic wet cough. OP-3957 II1 additionally reported neonatal RDS and presented reduced nNO-production rates (Table 1). Data on the fertility of *RSPH1*- and *RSPH9*-mutant males are so far, not reported. Here, we show in both *RSPH1*-mutant individuals affected male fertility due to asthenozoospermia, through a combination of an increased proportion of immotile and dysmotile sperm with non-progressive or slow-progressive patterns (Supplementary Videos S4, S5). OP-3957 II1 additionally presented a lowered sperm count and morphological abnormalities, showing the combination of oligoasthenoteratozoospermia (Table 1). *RSPH9*-mutant individual OP-2491 II1, who presented with chronic destructive lung disease, underwent a middle lobe resection of the lung, due to bronchiectasis. He also displayed reduction of the nNO-production rate and reported infertility due to dysmotile sperm cells (Supplementary Video S6 and Table 1).

**FIGURE 4:**
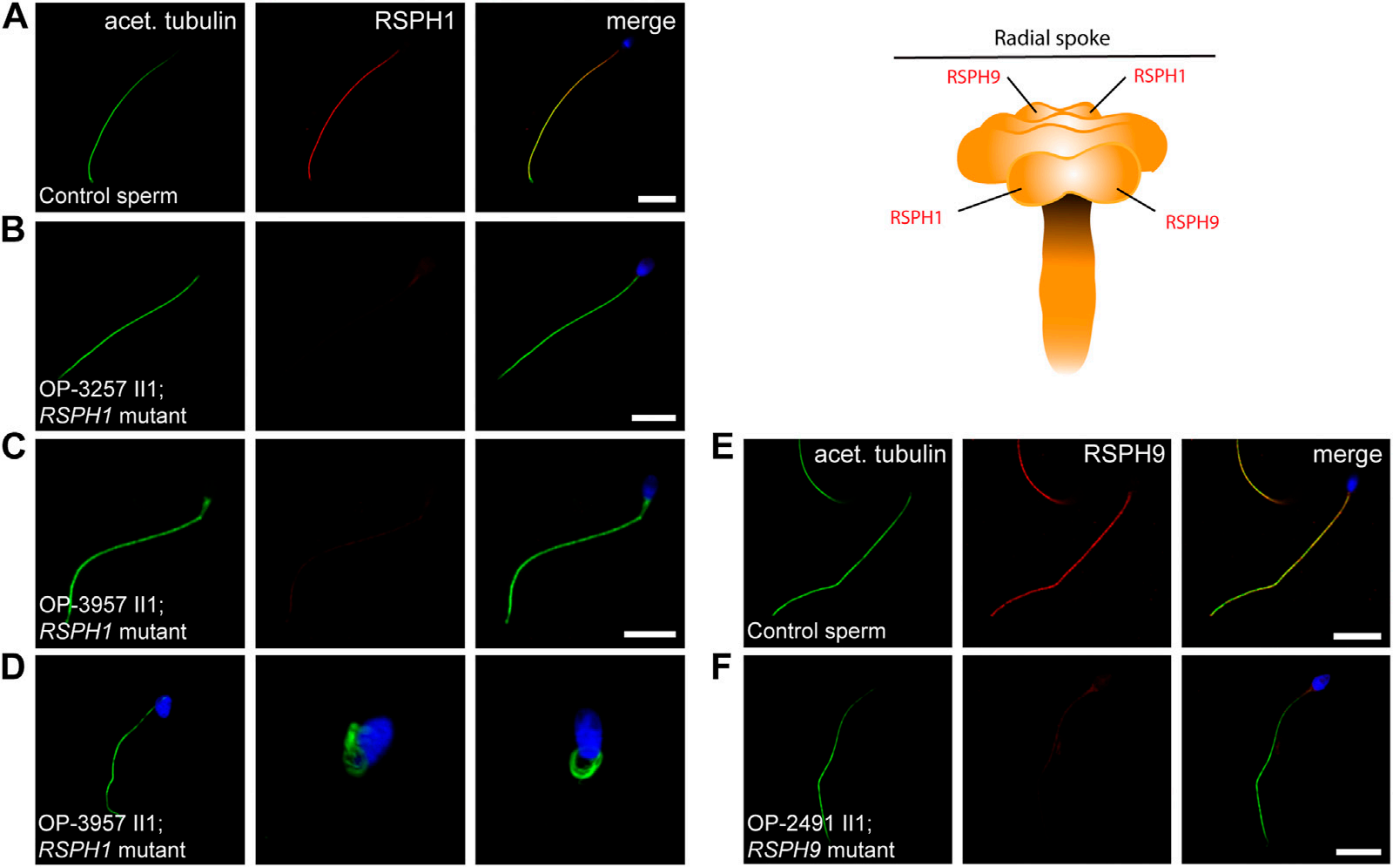
*RSPH1* and *RSPH9* mutations cause abnormal radial spoke head composition in sperm flagella. Sperm cells from healthy control donors and infertile male individuals with pathogenic variants in *RSPH1* and *RSPH9* were co-stained with antibodies directed against acetylated α-tubulin (acet. tubulin; depicted in green) and RSPH1 and RSPH9, respectively (both depicted in red). **(A)** In control sperm cells, immunoreactivity against RSPH1 (red) is observed along the entire flagellar length. The RSPH1 signal co-localizes with the flagellar marker acet. tubulin (depicted in yellow in the merged image). **(B,C)** In sperm flagella of *RSPH1*-mutant individuals OP-3257 II1 and OP-3957 II1, a severe reduction of the RSPH1 signal or no signal is observed along the flagellar axonemes. **(D)** Sperm cells of *RSPH1*-mutant individual OP-3957 II1 display morphological abnormalities, comprising bent flagella and sperm head defects consistent with MMAF. **(E)** In control sperm cells, RSPH9 (red) shows a panaxonemal staining pattern and co-localization with the flagellar marker acet. tubulin (depicted in yellow in the merged image). **(F)** In contrast, sperm flagella of the *RSPH9*-mutant individual OP-2491 II1 display a severe reduction of the RSPH9 signal. The schematic shows the putative localization of the RSPH1 and RSPH9 proteins in the radial spoke head complex. Nuclei were stained with Hoechst33342. Scale bars represent 10 µm.

We have previously demonstrated by IF microscopy in respiratory cilia the absence of RSPH1 and RSPH9 from *RSPH1*- and *RSPH9*-mutant cilia, respectively, and thus confirmed pathogenicity of the identified variants (Frommer et al., 2015) (Supplementary Figure S3). The protein composition of RS heads in sperm flagella has not been studied, so far. To characterize it in more detail, we tested here by IF microscopy whether the RS head composition is affected in *RSPH1*- and *RSPH9*-mutant sperm flagella. In *RSPH1*-mutant individuals OP-3257 II1 and OP-3957 II1 we examined the sperm flagellar localization of RSPH1. In healthy control samples, RSPH1 localizes along the entire flagellar length and co-localizes with the flagellar marker acetylated α-tubulin (Figure 4A). *RSPH1*-mutant sperm flagella displayed no signal against the RSPH1 protein (Figures 4B, C), consistent with the detected loss-of-function mutations. RSPH9 also displayed a panaxonemal localization pattern in control sperm flagella (Figure 4E). As expected, in sperm cells of *RSPH9*-mutant individual OP-2491 II1 RSPH9 was either severely reduced or absent in the flagellar axonemes (Figure 4F). Thus, our findings demonstrate for the first time that the RS head proteins RSPH1 and RSPH9 are also present in sperm flagella. In addition, we demonstrate that the pathogenicity of *RSPH1* and *RSPH9* variants can be also established by IF analyses in sperm cells. This is of particular interest, because the *RSPH9*-mutant individual carried a homozygous missense variant.

**FIGURE 5:**
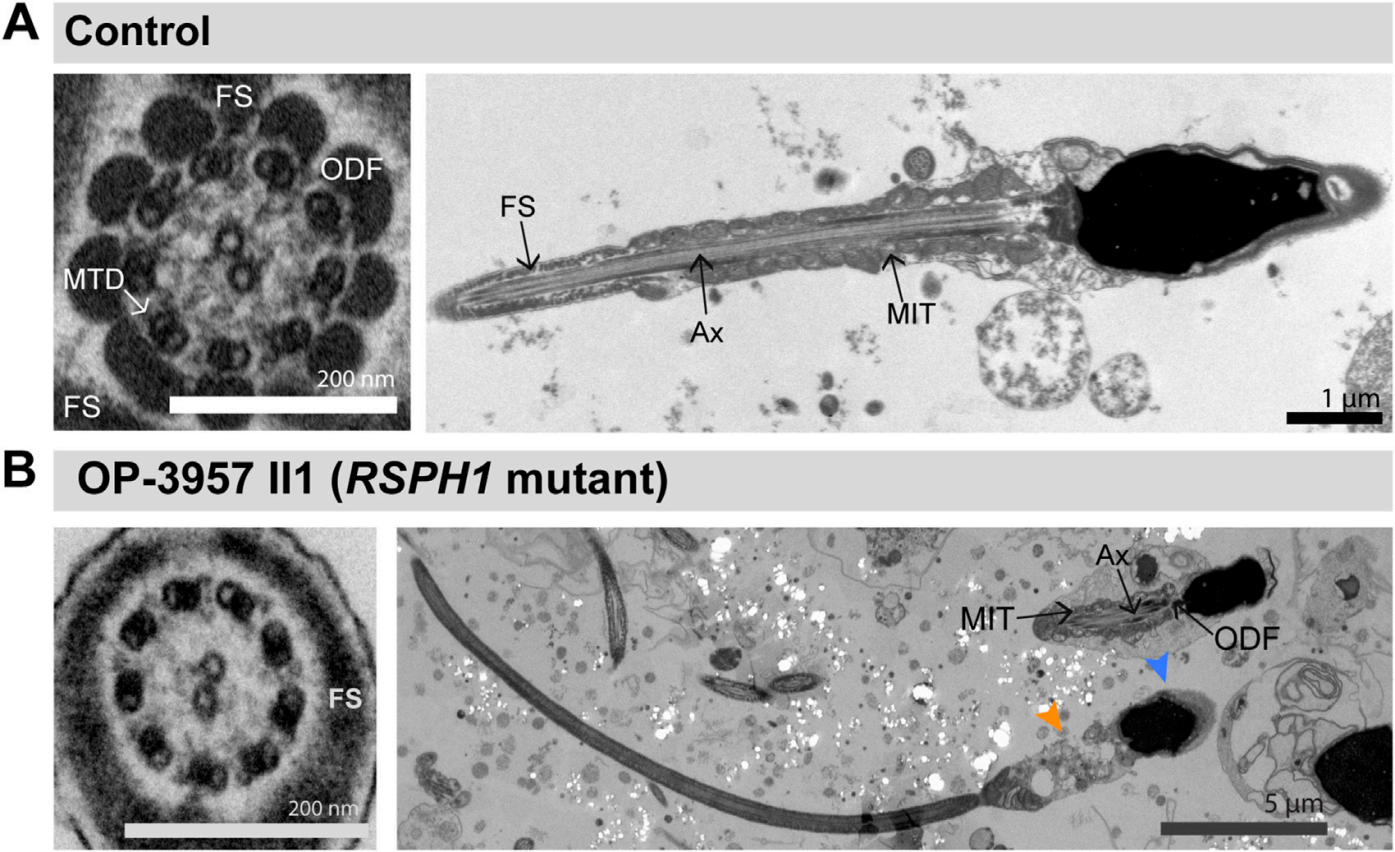
TEM analyses of *RSPH1*-mutant sperm cells displayed morphological abnormalities of the midpiece and head region, but preservation of the 9 + 2 axonemal organization. **(A)** TEM micrographs of sperm samples from a healthy control donor, showing a flagellar cross section with a regular 9 + 2 axoneme of microtubule doublets (MTD), (left image), and a longitudinal section, with regularly arranged mitochondria (MIT) and fibrous sheath (FS), respectively in the flagellar mid piece and principal piece (right image). **(B)** TEM micrographs of sperm samples from the *RSPH1*-mutant individual OP-3957 II1 show morphological abnormalities with dysplasia of the flagellar midpiece and abnormal head shapes (respectively indicated by orange and blue arrowheads), or sperm with only a cytoplasmic bag containing unassembled axonemal (Ax) and peri-axonemal components, such as outer dense fibers (ODF) and MIT (right image). Some sperm flagellar cross sections also display a normal ultrastructure with a regularly disposed 9 + 2 axoneme (left image).

Because the *RSPH1*-mutant individual OP-3957 II1 displayed abnormal sperm morphology in semen and IF microscopy analyses (Figure 4), we further examined this finding on the ultrastructural level. TEM micrographs of *RSPH1*-mutant sperm cells from OP-3957 II1 confirmed morphological defects of the sperm flagella in the mid piece and head region. Notably, as typically observed in MMAF, TEM micrographs showed sperm with cytoplasmic bags containing not fully assembled axonemal structures (Figure 5). Interestingly, the majority of flagellar cross sections displayed normal ultrastructure with a regularly disposed 9 + 2 axoneme resembling findings previously reported in *RSPH1*-mutant respiratory ciliary axonemes (Frommer et al., 2015) (Figure 5). Thus, both TEM and IF analyses confirmed presence of sperm cells with typical MMAF phenotypes, which has not been previously reported.

### Pathogenic variants in *SPEF2* and *HYDIN* cause MMAF-related infertility and absence of the central pair associated protein SPEF2 from flagellar axonemes

We and others have reported male infertility in *HYDIN*-and *SPEF2*-mutant individuals (Olbrich et al., 2012; Sha et al., 2019). So far, the molecular defects in sperm flagella of *HYDIN*-mutant males have not been characterized previously. Here, we had the opportunity to address this important research question because we identified three males with pathogenic variants in *SPEF2* and *HYDIN,* encoding components of the central pair associated protein complexes C1b and C2b, respectively. For individual OP-3022 II1, we detected the homozygous transition c.6140C>G (p.Ser 2047*) in *HYDIN* (CCDS59269; NM_001270974.2), leading to a premature translational stop, that segregates within the family (Supplementary Figure S1C). This variant is classified as likely pathogenic (class 4) by ACMG criteria. In individuals SP-24 and SP-32, we identified compound heterozygous pathogenic variants in *SPEF2* (CCDS43309; GenBank: NM_024867.4) (Supplementary Figure S1D). In individual SP-24, we identified variants c.910C>T (rs758170951) (p.Arg304*) and c.2629del (p.Ile877Phefs*Ter6), leading to a premature stop of translation and a frameshift with translational stop, respectively (Supplementary Figure S1D). These variants are classified as pathogenic (class 5) and likely pathogenic (class 4) by ACMG criteria, respectively. In Individual SP-32, we identified the frameshift-stop variant c.2763_2764del (p.Glu921Aspfs*31) and the missense variant c.3620G>A (rs200722554) (p.Arg1207Lys) (Supplementary Figure S1D). According to the ACMG guidelines, this missense variant is classified as of uncertain significance (class 3), whereas the loss-of-function variant is classified as likely pathogenic (class 4). Consistent with an autosomal recessive inheritance pattern, Sanger Sequencing confirmed segregation of *SPEF2* variants within the families (Supplementary Figure S1D).

Individual OP-3022 II1 with pathogenic variants in *HYDIN* reported neonatal RDS and was diagnosed with chronic sinusitis, otitis media and wet cough, and a very low nNO-production rate. He presented with infertility and semen analyses revealed dysmotile (Supplementary Video S7) and morphologically abnormal sperm, thus asthenoteratozoospermia (Table 1).

*SPEF2*-mutant individuals SP-24 and SP-32 presented with male infertility and did not have a prior diagnosis of any chronic airway disease. Both were diagnosed with dysmotile sperm cells (Supplementary Videos S8, S9) showing morphological abnormalities (asthenoteratozoospermia). Moreover, both individuals reported chronic upper airway symptoms including severe sinusitis (Table 1), consistent with PCD. For the characterization of sperm flagellar defects associated with the CP associated complexes C1b and C2b, we additionally included *HYDIN*-mutant individual OP-305 II2. The genetic diagnosis and clinical characteristics of OP-305 II2 were reported previously (Olbrich et al., 2012) and are summarized in Table 1.

We have previously shown in respiratory cilia that IF analyses using antibodies directed against SPEF2 can be applied to diagnose and/or confirm the effect of genetic variants in the CP-apparatus related (C1b and C2b) genes *SPEF2* and *HYDIN*, because the C1b protein SPEF2 is absent from respiratory ciliary axonemes (Cindrić et al., 2019). Here, we confirmed absence of SPEF2 from respiratory ciliary axonemes in the *HYDIN*- and *SPEF2*-mutant individuals OP-3022 II2, SP-24 and SP-32 (Supplementary Figure S4). Next, we tested whether SPEF2 localization also depends on functional HYDIN in sperm cells.

**FIGURE 6:**
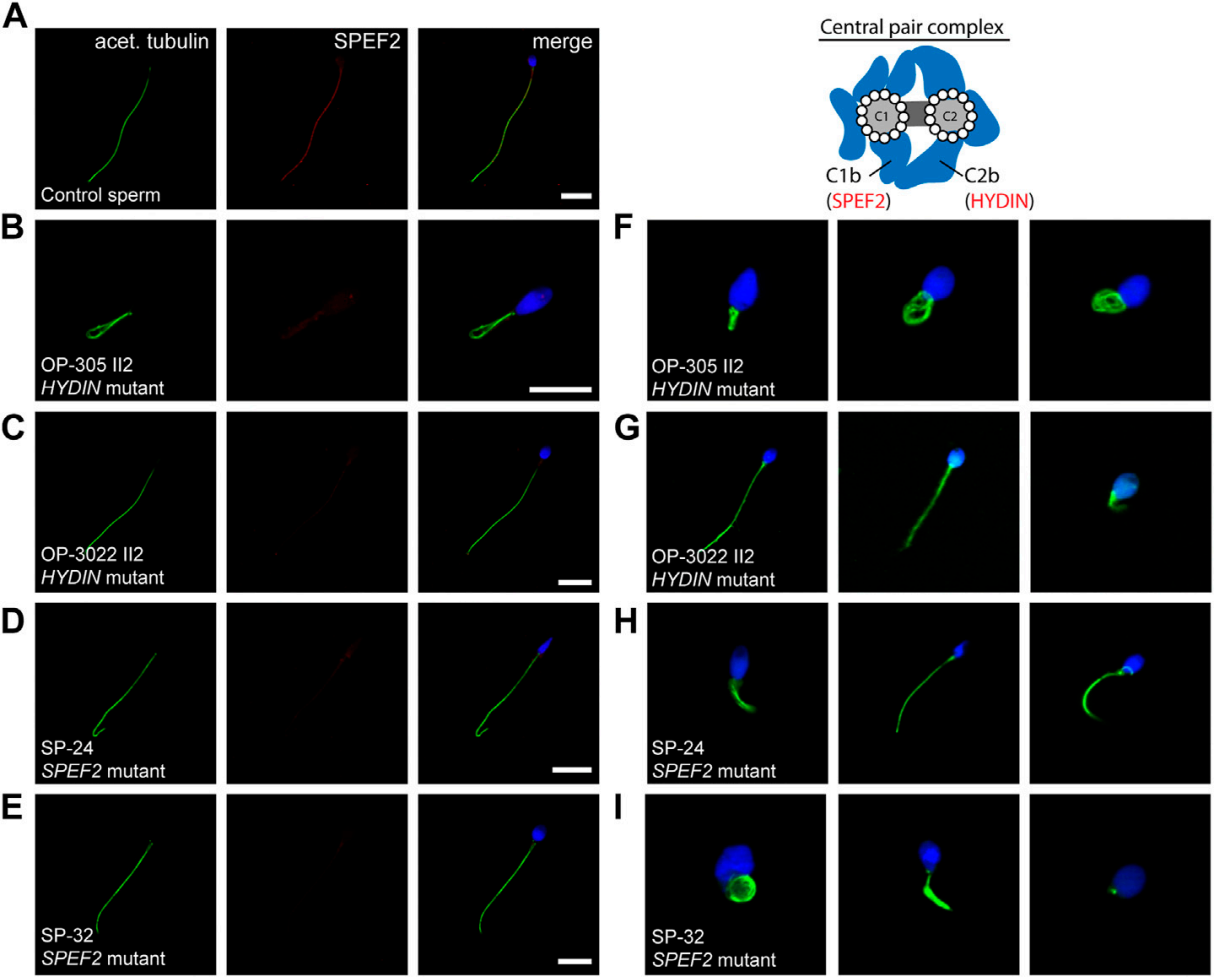
SPEF2 is absent or severely reduced in sperm flagellar axonemes of *HYDIN*- and *SPEF2*-mutant individuals, presenting with MMAF-associated infertility. Sperm cells from healthy control donors and infertile male individuals with pathogenic variants in *HYDIN* and *SPEF2* were co-stained with antibodies directed against acetylated α-tubulin (acet. tubulin; depicted in green) and SPEF2 (depicted in red). **(A)** In control sperm cells, immunoreactivity against SPEF2 (red) is observed along the entire flagellar length. The SPEF2 signal co-localizes with the flagellar marker acet. tubulin (depicted in yellow in the merged image). **(B–E)** In sperm cells of both *HYDIN*-mutant individuals (OP-305 II2 and OP-3022 II2) and *SPEF2*-mutant individuals (SP-24 and SP-32), a severe reduction of the SPEF2 signal or no signal against SPEF2 was observed along the flagellar axoneme. **(F–I)** Sperm of both *HYDIN*-and *SPEF2*-mutant individuals display a MMAF phenotype with short, bent, coiled and irregular flagella. The schematic representatively shows the localization of HYDIN and SPEF2 within the central pair associated apparatus. Nuclei were stained with Hoechst33342. Scale bars represent 10 µm.

In control sperm cells we observed immunoreactivity against SPEF2 along the entire flagellar length, that co-localizes with the flagellar marker acetylated α-tubulin (Figure 6A). Consistent with findings in respiratory cilia, sperm cells of *HYDIN*-mutant individuals OP-305 II2 and OP-3022 II2, as well as SPEF2-mutant individuals SP-24 and SP-32, all displayed an absent or severely reduced signal against SPEF2 along the entire flagellar length (Figures 6B–E). Moreover, IF microscopy analyses clearly showed the presence of morphological sperm abnormalities in all affected individuals, typical for a MMAF phenotype, with short, bent, curled and irregular flagella (Figures 6F–I).

Our IF analyses show that HYDIN and SPEF2 also interact within the CP apparatus of sperm flagella, which has not been previously reported. Thus, IF microscopy in sperm flagella with antibodies targeting SPEF2 can help to determine pathogenicity of DNA variants in *HYDIN*- and *SPEF2*-mutant individuals, aiding diagnosis of male infertility. This is of particular interest because one of the *SPEF2*-mutant individuals harbored missense variants with uncertain pathogenicity.

**FIGURE 7:**
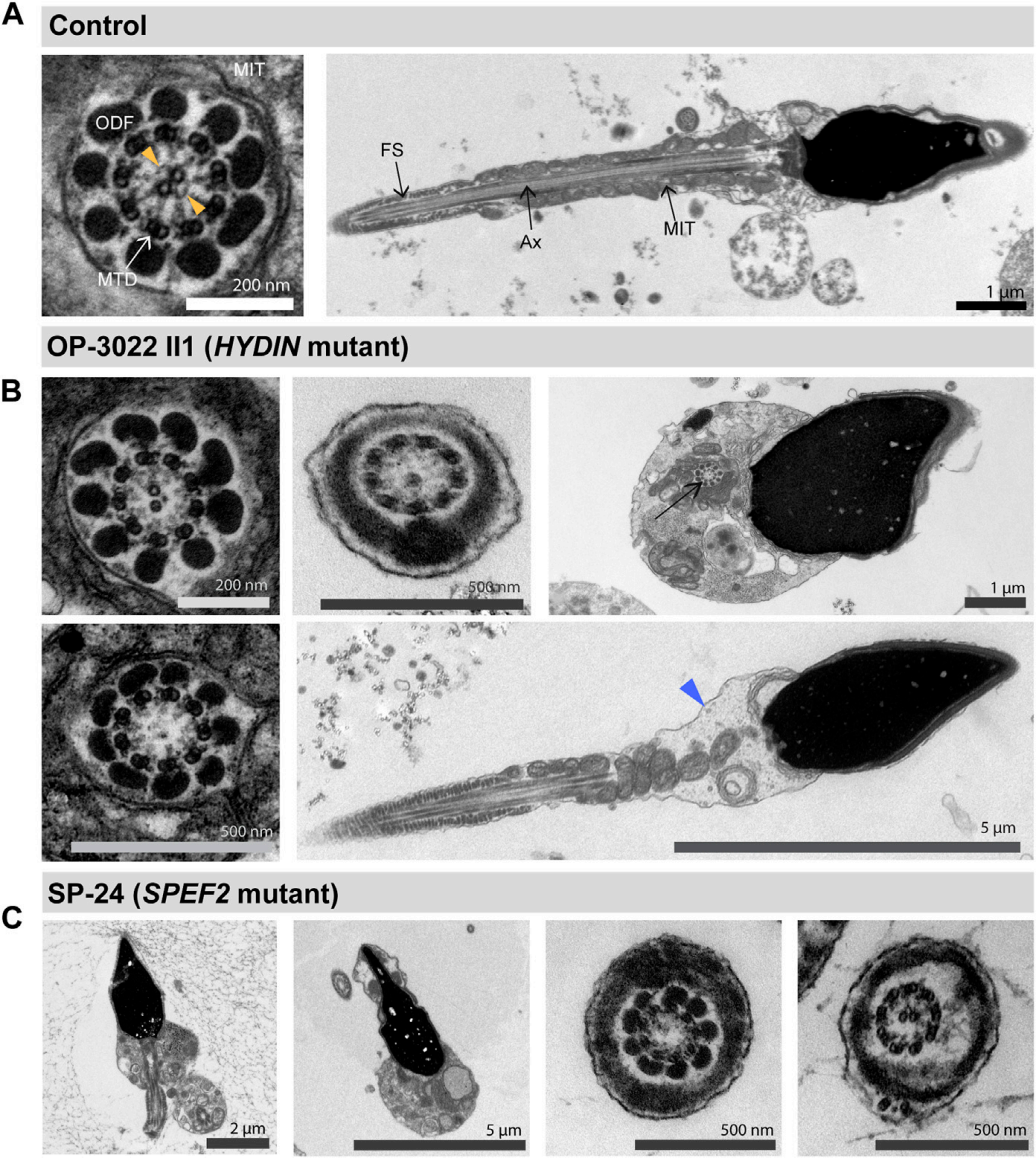
TEM analyses of HYDIN and SPEF2-deficient sperm displays morphological abnormalities. **(A)** TEM micrographs of sperm samples from a healthy control donor, showing a flagellar cross section with a regular 9 + 2 axoneme of microtubule doublets (MTD), (left image), and a longitudinal section showing the axoneme (Ax) at the center, with regularly arranged mitochondria (MIT) and fibrous sheath (FS), respectively in the flagellar mid piece and principal piece (right image). The orange arrowheads in the cross section indicate projections of the central pair associated apparatus. **(B)** *HYDIN*-mutant sperm display flagellar cross sections with either a normal 9 + 2 ultrastructure of the axoneme displaying less pronounced CP projections (upper left image), or 9 + 1 cross sections with only one central microtubule (upper middle image), or a 9 + 0 axoneme without the CP (lower left image). Morphological abnormalities of the sperm flagella are also observed, comprising sperm cells displaying a cytoplasmic bag with unassembled axonemal and peri-axonemal components (upper right image, arrow), or mid piece dysplasia (lower right image, indicate by a blue arrowhead). **(C)** *SPEF2*-mutant sperm cells also display short and irregular flagella (first left image), or a cytoplasmic bag with unassembled flagellar components (second left image). Flagellar cross sections display either a 9 + 0 structure without the CP (second from the right image), or a 9 + 2 structure (first from the right image).

We confirm MMAF phenotypes in *SPEF2*-mutant male individuals SP-24 and SP-32 (Figure 6), as previously reported (Liu et al., 2019; Sha et al., 2019; He et al., 2020; Tu et al., 2020). Interestingly, we now also observed MMAF phenotypes associated with pathogenic *HYDIN* variants (Figure 6), which has not been previously reported. However, this is not unexpected because we demonstrate that the large C1b protein SPEF2 is absent from the flagellar axonemes of *HYDIN*- and *SPEF2*-mutant individuals. To further characterize and confirm the flagellar defect at the ultrastructural level, we performed TEM studies of *HYDIN-* and *SPEF2-*mutant sperm from OP-3022 II2 and SP-24, respectively. TEM micrographs confirmed morphological sperm defects, including dysplasia of the mid piece, morphological head abnormalities, and the presence of sperm cells with stub flagella. Moreover, flagellar cross sections in both individuals displayed a combination of normal 9 + 2 ultrastructure, and altered 9 + 1 or 9 + 0 axonemes, retaining a single central microtubule or lacking the entire CP, respectively (Figure 7).

## Discussion

Male infertility is frequently observed in motile ciliopathies, such as PCD. Due to the evolutionarily conserved composition of the axoneme in motile cilia and sperm flagella, deficiencies can present in both cell types and also cause flagellar dysfunction (Wallmeier et al., 2020). However, cell-type specific compositions have been reported and explain cell-type specific disease affecting either sperm flagella or motile cilia. For example, mutations in the genes encoding the ODA β-HC paralogues *DNAH9* and *DNAH11* cause solely PCD and no defects or dysfunction of sperm flagella due to exclusive expression in motile cilia (Dougherty et al., 2016; Loges et al., 2018; Whitfield et al., 2019), whereas mutations of the gene encoding the ODA β-HC *DNAH17* cause MMAF due to exclusive expression in sperm cells (Whitfield et al., 2019). Consequently, genetic defects that are specific to motile cilia (e.g. *DNAH5*, *DNAH9*, *DNAH11* mutations) are not expected to affect sperm flagella but can cause fertility issues through dysfunction of efferent duct motile cilia in the male reproductive system (Aprea et al., 2021a).

Here, we investigated how genetically distinct defects of the axonemal rulers, RSPH1/RSPH9 and C1b/C2b complexes (Figure 1B) affect sperm flagella composition and function in comparison to respiratory cilia, revealing a high conservation of these axonemal complexes in both motile cilia and sperm cells.

Our findings show that mutations in *CCDC39* and *CCDC40* result in the absence of CCDC39 from sperm flagella axonemes (Figure 2). This is consistent with previous data obtained in ciliated respiratory epithelial cells, where an interdependence of both axonemal ruler components was observed, due to the absence of CCDC39 in both *CCDC39*- and *CCDC40*-mutant individuals affected by PCD (Becker-Heck et al., 2011; Merveille et al., 2011; Antony et al., 2013). More recently, both genes were also associated to MMAF-related male infertility and the absence of CCDC39 was observed in *CCDC39*-mutant sperm flagella (Chen et al., 2021; Xu et al., 2022). Our results confirm these findings. HVMA, IF microscopy and TEM of sperm samples from OP-122 (*CCDC39*-mutant) and OP-1516 II1 (*CCDC40*-mutant) show immotile sperm cells with a typical MMAF phenotype of short, bent and irregular flagella (Supplementary Videos S2, S3; Figure 3). Of note, our findings showed for the first time that IF microscopy with antibodies directed against CCDC39 can be used to identify also *CCDC40*-mutant sperm flagella (Figure 2). This is of particular interest for diagnostic purposes, as certain genetic analyses can be inconclusive if variants of unknown significance are found. In those cases, protein analyses documenting a defect can qualify interpretation of the genetic results as pathogenic. Interestingly, sperm flagella cross sections of *CCDC40*-mutant individual OP-1516 II1 displayed a tubular disorganization, as previously reported in CCDC39 and CCDC40 deficient respiratory cilia (Becker-Heck et al., 2011; Merveille et al., 2011; Antony et al., 2013), further confirming the functional preservation of the axonemal ruler in both cell types.

Particularly for individuals affected by RS head dysfunction, no data has been reported on the impact of male fertility. Here, we reported the fertility status of three individuals, two of them carrying pathogenic variants in *RSPH1* and one in *RSPH9*. All presented with associated infertility due to asthenozoospermia (Supplementary Videos S4–S6) with (OP-3957 II1) or without (OP-3257 II1 and OP-2491 II1) morphological abnormalities of the sperm (Figures 4, 5), providing novel evidence for the functional conservation of both RSPH1 and RSPH9 also in sperm flagella.

In ciliated respiratory epithelial cells, IF microscopy is predominantly used for the diagnosis of RS head defects (Frommer et al., 2015). Other tests, such as HVMA and TEM, are less indicative for an underlying defect. Sperm flagella of *RSPH1*- and *RSPH9*-mutant individuals display a clear motility defect in HVMA, however TEM cross sections of sperm flagella from OP-3957 II1 (*RSPH1*-mutant) display a normal 9 + 2 axonemal ultrastructure (Figure 5). By using IF microscopy, we observed absence of RSPH1 and RSPH9 from sperm flagella of *RSPH1*- and *RSPH9*-mutant individuals, respectively (Figure 4). Hereby, we confirmed that the dysmotility of sperm flagella is associated with the deficiency of the respective RS head components. In sperm flagella, IF stainings directed against RSPH1 and RSPH9 were so far only reported in sperm flagella of humans and mice with other genetic defects (Tu et al., 2020; Shen et al., 2021; Zhang et al., 2021). Here, we show anti-RSPH1 and anti-RSPH9 stainings in both respiratory cilia and sperm flagella from the same individuals with corresponding pathogenic gene variants. This underlines the specificity of IF microscopy also in the diagnosis of RS head defects in sperm, which might be especially useful in individuals harboring variants of unknown significance in order to help interpretation of genetic findings (e.g., in OP-2491 II1 carrying homozygous missense variant c.244T>C).

Interestingly, in *CCDC40*-mutant individual OP-1516 II1 and *RSPH1*-mutant individual OP-3957 II1, reduced sperm counts were additionally diagnosed. We previously reported obstructive oligozoospermia (reduced sperm count) in DNAH5 deficient human men and male mice, caused by dysmotility of efferent duct cilia (Aprea et al., 2021a). It is possible that CCDC40 and RSPH1 deficiencies also cause dysmotility of efferent duct cilia by impairing the passage of sperm from the testis to the epididymis. In contrast to axonemal ODAs, which present cell type specific paralogs and thus cell type specific defects, CCDC40 and RSPH1 deficiencies are expected to affect male fertility by two mechanisms, because both sperm flagella and motile cilia of the efferent ducts are defective. However, in this study, we found decreased sperm counts in only two affected individuals, limiting the reproducibility required to confirm this hypothesis. It would be interesting to study this hypothesis in a larger cohort and in model organisms such as mice.

Defects of the CP associated apparatus are difficult to diagnose. They are not associated with situs abnormalities and diagnostic tests in respiratory cilia, such as TEM are inconclusive and HSVM is difficult to interpret (Olbrich et al., 2012; Cindrić et al., 2019; Raidt et al., 2022). The CP complex associated genes *HYDIN* and *SPEF2* are also associated with male infertility due to asthenoteratozoospermia (Olbrich et al., 2012; Cindrić et al., 2019; Sha et al., 2019). Our findings confirmed that the motility (Supplementary Video S7–S9) and ultrastructure (Figure 7) of *HYDIN*- and *SPEF2*-mutant sperm is strongly affected. Due to the presence of the expressed pseudogene *HYDIN2* with almost identical intron/exon structure to *HYDIN*, the generation of an antibody specifically targeting HYDIN is difficult (Cindrić et al., 2019). We have shown that IF analyses of respiratory cells using antibodies directed against SPEF2 can identify both *HYDIN*- and *SPEF2*-mutant respiratory cells, as axonemal SPEF2 localization depends on the presence of functional HYDIN and *vice versa*. While SPEF2 is reported to be absent in flagella of *SPEF2*-mutant sperm from MMAF affected males (Liu et al., 2019; Sha et al., 2019; He et al., 2020; Tu et al., 2020), it was not clear whether IF stainings using antibodies directed against SPEF2 could also detect HYDIN defects in sperm flagella. Here, we now demonstrate that SPEF2 is absent not only from sperm flagella of *SPEF2*-mutant individuals but also from those of *HYDIN*-mutant individuals (Figure 6). These findings provide new evidence for an interaction of SPEF2 and HYDIN also in sperm flagella and corroborate that the IF analysis using antibodies against SPEF2 is also suitable for the identification of HYDIN defects in sperm flagella. This is especially important, because the interpretation of genetic variants in *HYDIN* is confounded by sharing most of its DNA sequence to the pseudogene *HYDIN2*.

Taken together, our results indicate that the ruler proteins CCDC39 and CCDC40, the radial spoke head proteins RSPH1 and RSPH9 as well as the central pair associated apparatus proteins SPEF2 and HYDIN have a similar functional role in motile cilia and sperm flagella, as hypothesized in Figure 1. In addition, our study demonstrates that IF microscopy analyses are valuable diagnostic tools for the identification of defects of the axonemal composition of sperm flagella related to the axonemal rulers, radial spoke head and the central pair associated apparatus C1b and C2b. This knowledge is especially helpful in cases where DNA variants of unknown significance are found by gene testing.

Interestingly, some men were recruited primarily from the andrology department because of infertility problems and did not receive a pneumology work up examination until this study. In all of these individuals, we were able to identify also respiratory disease (Table 1), as it was reported for genetic mutations in e.g., *SPEF2*, which cause severe lung disease besides the MMAF phenotype in affected male individuals (Cindrić et al., 2019). The results of our study suggest that critical investigation of other disease manifestations in infertile men, particularly respiratory, is of great importance and should be considered especially in men with mutations in specific genes. In particular, affected individuals with e.g., lung disease will clearly benefit from early and appropriate treatment.

## Data Availability

The datasets presented in this study can be found in online repositories. The names of the repository/repositories and accession number(s) can be found in the article/Supplementary Material.
