# Supplementary material for: Pathogenic gene variants in CCDC39, CCDC40, RSPH1, RSPH9, HYDIN, and SPEF2 cause defects of sperm flagella composition and male infertility
Source: Front Genet. 2023 Feb 17;14:1117821. doi: 10.3389/fgene.2023.1117821 (PMC9981940; doi:10.3389/fgene.2023.1117821)
Supplement: Supplementary file 10 [file DataSheet1.docx]

### Supplementary Material


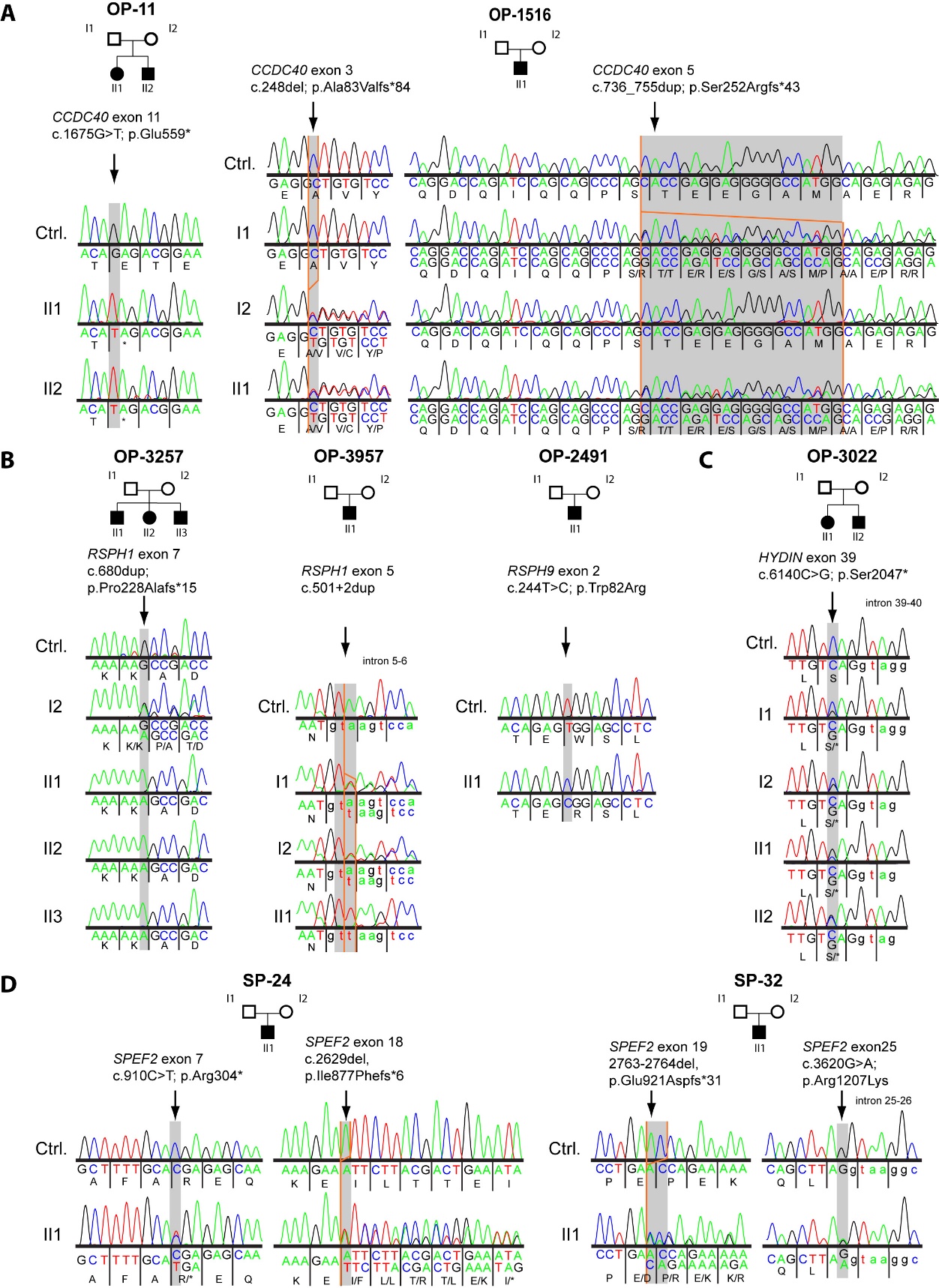


**Supplementary Figure 1: Pedigrees of study participants and verification of pathogenic variants by Sanger Sequencing.**

**(A)**: Sanger sequencing of families with pathogenic variants in axonemal ruler complex associated gene CCDC40. In family OP-11, a homozygous stop variant in exon 11 was confirmed in index individual OP-11 II2 and its sibling OP-11 II1 (c.1675G>T; p.Glu559*). In family OP-1516 compound heterozygous variants in exon 3 (c.248del; p.Ala83Valfs*84) and exon 5 (c.736_755dup; p.Ser252Argfs*43) were confirmed in index individual OP-1516 II1. Sequencing results reveal that both variants segregate. The variant c.248del in exon 3 was inherited by the mother OP-1516 I2, and variant c.736_755dup in exon 5 by the father OP-1516 I1. **(B)**: Sanger sequencing of families with pathogenic variants in RS head associated genes RSPH1 and RSPH9. In individual OP-3257 II1 and its siblings we confirm homozygous frameshift stop variants in RSPH1 exon 7 (c.680dup; p.Pro228Alafs*15). In the mother OP-3257 I2 the variant is detected only in a heterozygous state. In family OP-3957 we confirm the splice-site variant c.501+2dup in RSPH1 intron 5-6. Index individual OP-3957 II1 is affected in a homozygous state, whereas in the parents OP-3957 I1 and I2 the variant is detected only in a heterozygous state. In individual OP-2491 II1 we confirm the homozygous missense variant c.244T>C (p.Trp82Arg) in RSPH9 exon 2. **(C)**: Sanger sequencing of family OP-3022 with pathogenic variants in CP-apparatus associated gene HYDIN. We confirm the stop variant (c.6140C>G; p.Ser2047*) in exon 39. Due to the autosomal recessive inheritance pattern of HYDIN and the reported clinical symptoms, affected index individual OP-3022 II2 and its affected sibling are predicted to be homozygous for this stop variant. Due to the presence of the pseudogene HYDIN2, that is almost identical in exon/intron structure to HYDIN, genetic testing is difficult. This can be observed in the electropherograms of family OP-3022, where the detected gene variant seems to be heterozygous also in the affected siblings. **(D)**: Sanger sequencing of individuals SP-24 and SP-32 with pathogenic variants in CP-apparatus associated gene SPEF2. In SP-24 we confirm compound heterozygous loss-of-function variants in exon 7 (c.910C>T; p.Arg304*) and exon 18 (c.2629del; p.Ile877Phefs*6). In SP-32 we also confirm pathogenic compound heterozygous variants. A frameshift stop variant in exon 19 (c.2763-2764del; p.Glu921Aspfs*31) and a missense variant in exon 25 (c.3620G>A; p.Arg1207Lys).


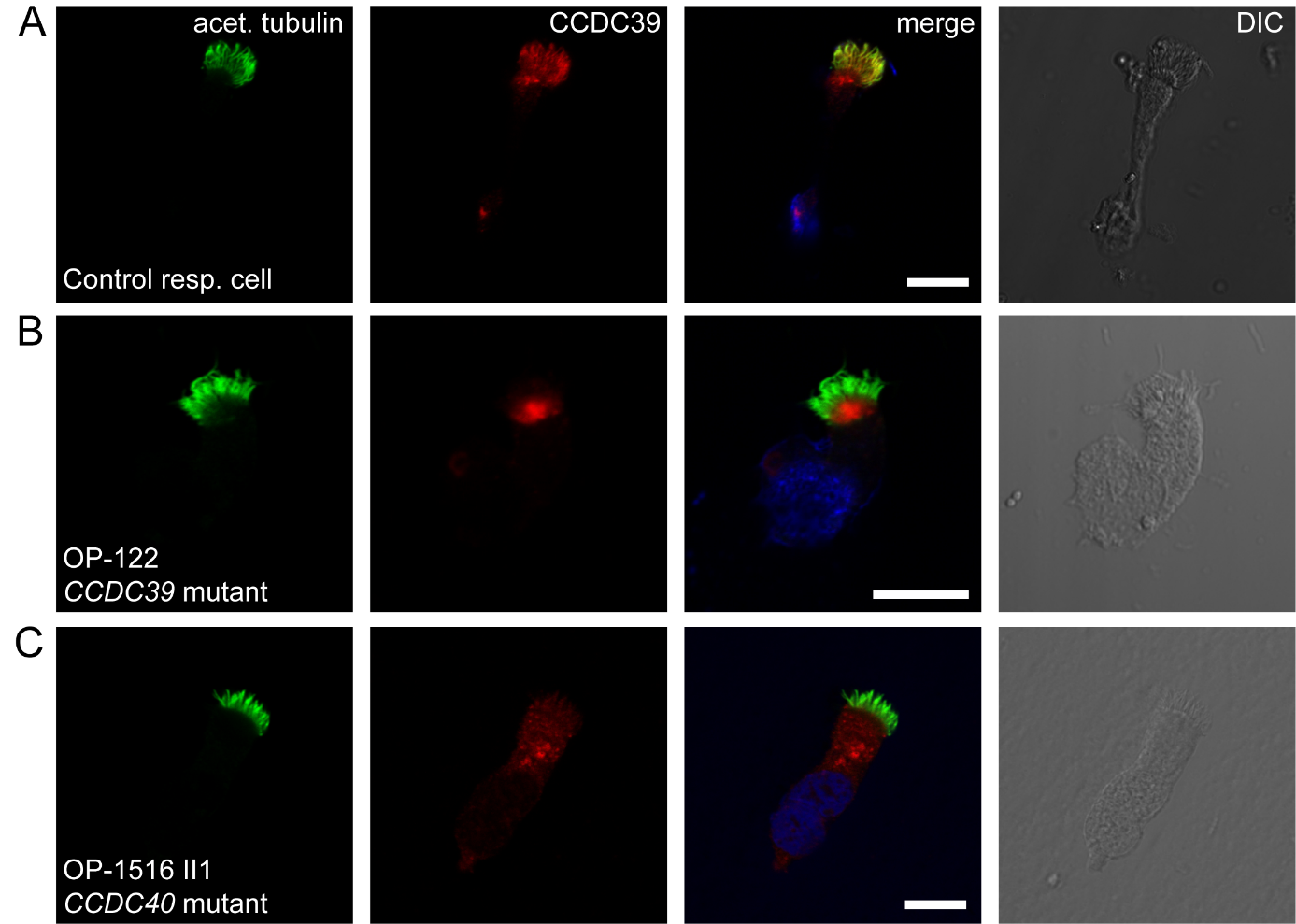


**Supplementary Figure 2: CCDC39 is absent along ciliary axonemes of CCDC39- and CCDC40-mutant individuals.** Ciliated respiratory epithelial cells from healthy control donors and male individuals with pathogenic variants in CCDC39 and CCDC40 were co-stained with antibodies directed against acetylated α-tubulin (acet. tubulin; depicted in green) and CCDC39 (depicted in red). **(A)** In control cilia, CCDC39 (red) localizes along the entire ciliary axonemal length and co-localizes with the ciliary marker acet. tubulin (depicted in yellow in the merged image). **(B and C)** In respiratory cilia of CCDC39-mutant individual OP-122 and CCDC40-mutant individual OP-1516 II1, no signal against CCDC39 is observed. Nuclei were stained with Hoechst33342. Scale bars represent 10µm. **DIC**: differential interference contrast.


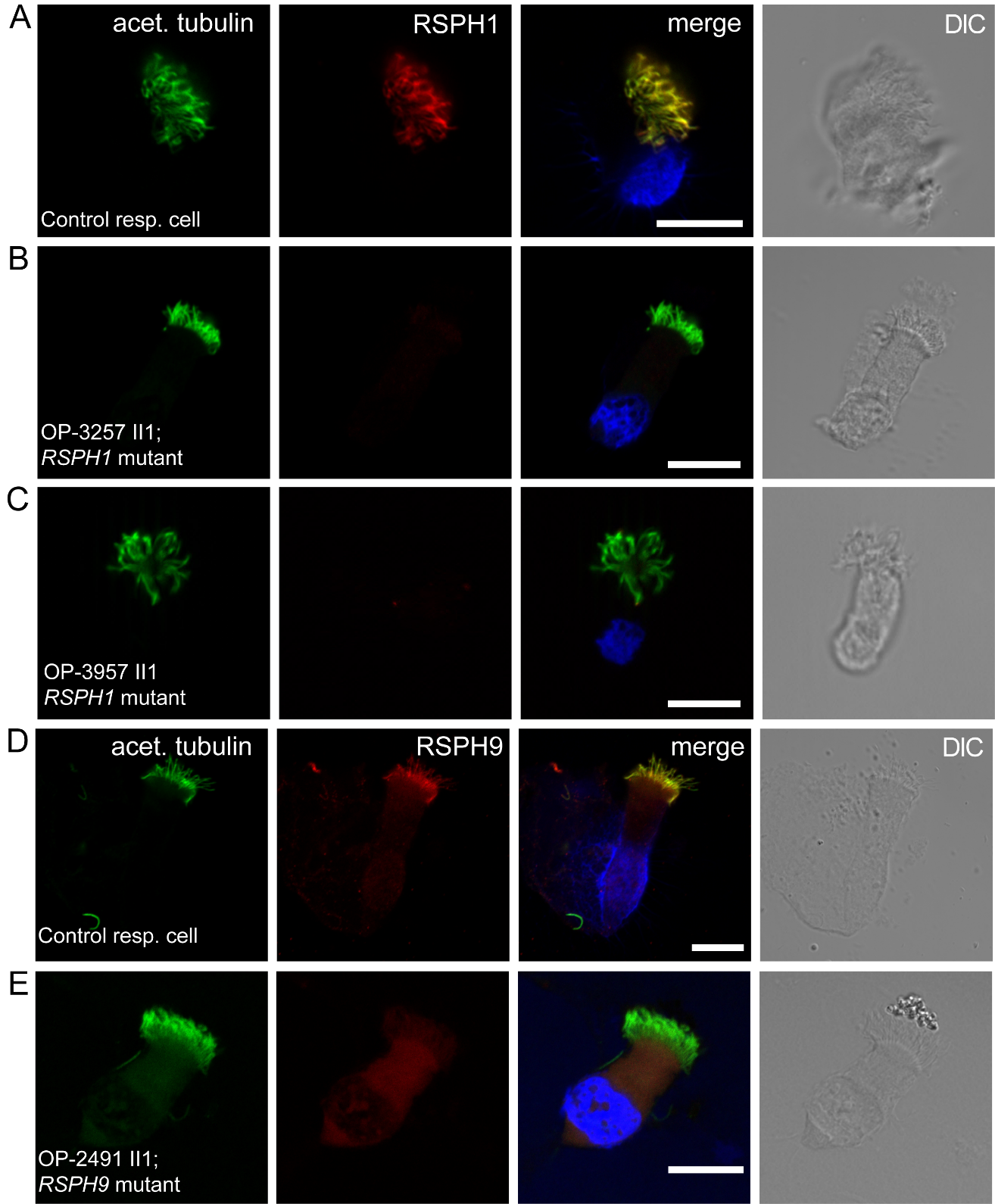


**Supplemental Figure 3: RSPH1 and RSPH9 are absent along ciliary axonemes of respectively RSPH1- and RSPH9-mutant individuals.** Ciliated respiratory epithelial cells from healthy control donors and males with pathogenic variants in RSPH1 and RSPH9 were co-stained with antibodies directed against acetylated α-tubulin (acet. tubulin; depicted in green) and respectively RSPH1 and RSPH9 (both depicted in red). **(A and D)** In control cilia, RSPH1 (A) and RSPH9 (D) localize along the entire ciliary axonemal length and co-localize with the ciliary marker acet. tubulin (depicted in yellow in the merged image). **(B and C)** In respiratory cilia of RSPH1-mutant individuals OP-3257 II1 and OP-3957 II1 no signal against RSPH1 is observed. **(E)** In respiratory cilia of RSPH9-mutant individual OP-2491 II1 no signal against RSPH9 is observed. Nuclei were stained with Hoechst33342. Scale bars represent 10µm. **DIC**: differential interference contrast.


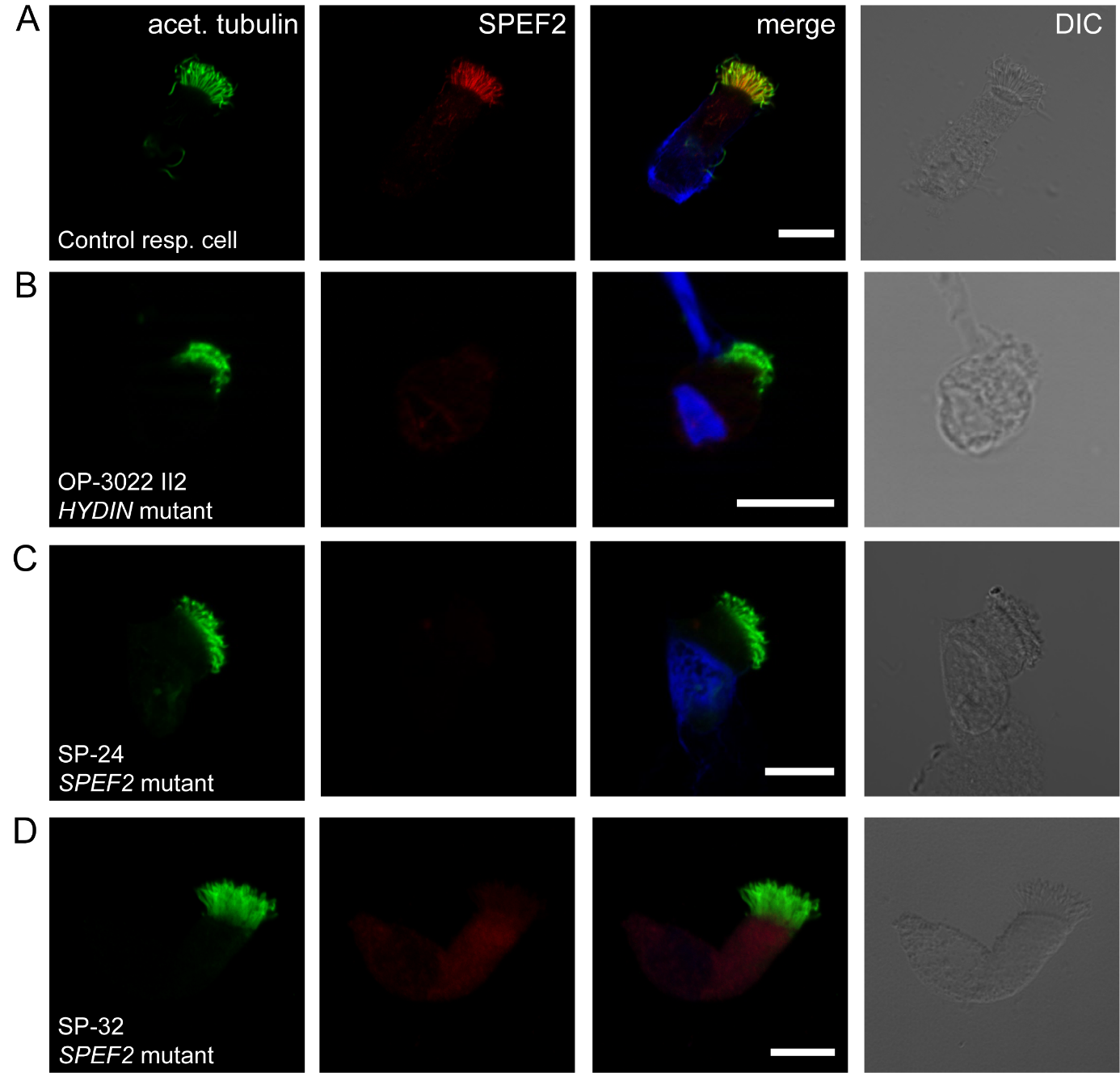


**Supplemental Figure 4: SPEF2 is absent along ciliary axonemes of HYDIN- and SPEF2-mutant individuals.** Ciliated respiratory epithelial cells from healthy control donors and males with pathogenic variants in HYDIN and SPEF2 were co-stained with antibodies directed against acetylated α-tubulin (acet. tubulin; depicted in green) and SPEF2 (depicted in red). **(A)** In control cilia, SPEF2 localizes along the entire ciliary axonemal length and co-localizes with the ciliary marker acet. tubulin (depicted in yellow in the merged image). **(B-D)** In respiratory cilia of HYDIN-mutant individual OP-3022 II2 and SPEF2-mutant individuals SP-24 and SP-32, no signal against SPEF2 is observed. Nuclei were stained with Hoechst33342. Scale bars represent 10 µm. **DIC**: differential interference contrast.

**Supplementary Table 1: Primer sequences used for Sanger Sequencing**

| **Primer name** | **Sequence (5’ 🡪 3’)** | **Variant to be verified** |
| --- | --- | --- |
| *CCDC40* Exon3 F | cctcacaaggtcctttatactttg | c.248delC |
| *CCDC40* Exon3 R | cccaagttcctcacataggg |  |
| *CCDC40* Exon5 F | tctgggatgcattgagtcag | c.736_755dup |
| *CCDC40* Exon5 R | accctacgaagaggcaggtc |  |
| *CCDC40* Exon11 F | tctgtgcattgagttcgcct | c.1675G>T |
| *CCDC40* Exon11 R | atccgtcttcctcctgagct |  |
| *HYDIN* Exon39 F | agtacccatgtaaattccgg | c.6140C>G |
| *HYDIN* Exon39 R | attgtgaatgccaacagtcatc |  |
| *RSPH1* Exon5 F | gcaccttctgactggacacag | c.501+2dupT |
| *RSPH1* Exon5 R | gtaccagaggctcagagaatgg |  |
| *RSPH1* Exon7 F | caactggtctgattcctgagtg | c.680dupA |
| *RSPH1* Exon7 R | atgtccacaactcccacagc |  |
| *RSPH9* Exon2 F | agtgggtgagaagggagttg | c.244T>C |
| *RSPH9* Exon2 R | agcccaggctctcttttagg |  |
| *SPEF2* Exon7 F | tggggtgaagcaaatgggat | c.910C>T |
| *SPEF2* Exon7 R | agtgacatttgatgcagagtagc |  |
| *SPEF2* Exon18 F | tggcagcctgtcttttcatt | c.2629delA |
| *SPEF2* Exon18 R | acaatgctttcagagagatggtc |  |
| *SPEF2* Exon19 F | ccaccactgccatcaaaagg | c.2763-2764delAC |
| *SPEF2* Exon19 R | gctactccgagggcttttcc |  |
| *SPEF2* Exon25 F | tcacaatgtcaagaggcacaga | c.3620G>A |
| *SPEF2* Exon25 R | ccctaataccacctgcagagtc |  |

**Supplementary Table 2: Antibodies and dilutions used in immunofluorescence stainings**

| **Antibodies** | **Manufacturer** | **Order number** | **Dilution** | |
| --- | --- | --- | --- | --- |
|  |  |  | **resp. cells** | **sperm** |
| Mouse monoclonal anti-acetylated α tubulin | Sigma Prestige Antibodies | T6793 | 1:10,000 | 1:4000 |
| Rabbit polyclonal anti-CCDC39 | Sigma Prestige Antibodies | HPA035364 | 1:200 | 1:200 |
| Rabbit polyclonal anti-RSPH1 | Sigma Prestige Antibodies | HPA016816 | 1:400 | 1:200 |
| Rabbit polyclonal anti-RSPH9 | Sigma Prestige Antibodies | HPA031703 | 1:100 | 1:100 |
| Rabbit polyclonal anti-SPEF2 | Sigma Prestige Antibodies | HPA039606 | 1:100 | 1:50 |
| Goat anti-mouse Alexa Fluor 488 | Thermo Fischer Scientific | A11029 | 1:1000 | 1:1000 |
| Goat anti-rabbit Alexa Fluor 546 | Thermo Fischer Scientific | A11035 | 1:1000 | 1:1000 |
